# The Trace Amine-Associated Receptor 1 Agonist 3-Iodothyronamine Induces Biased Signaling at the Serotonin 1b Receptor

**DOI:** 10.3389/fphar.2018.00222

**Published:** 2018-03-12

**Authors:** Julia Bräunig, Juliane Dinter, Carolin S. Höfig, Sarah Paisdzior, Michal Szczepek, Patrick Scheerer, Mark Rosowski, Jens Mittag, Gunnar Kleinau, Heike Biebermann

**Affiliations:** ^1^Institute of Experimental Pediatric Endocrinology, Charité - Universitätsmedizin Berlin, Corporate Member of Freie Universität Berlin, Humboldt-Universität zu Berlin, and Berlin Institute of Health, Berlin, Germany; ^2^Institute of Experimental Endocrinology, Charité - Universitätsmedizin Berlin, Corporate Member of Freie Universität Berlin, Humboldt-Universität zu Berlin, and Berlin Institute of Health, Berlin, Germany; ^3^Group Protein X-ray Crystallography and Signal Transduction, Institute of Medical Physics and Biophysics, Charité - Universitätsmedizin Berlin, Corporate Member of Freie Universität Berlin, Humboldt-Universität zu Berlin, and Berlin Institute of Health, Berlin, Germany; ^4^Center of Brain Behavior and Metabolism, University of Lübeck, Lübeck, Germany; ^5^Institute of Biotechnology, Department Medical Biotechnology, Technical University of Berlin, Berlin, Germany

**Keywords:** trace amine-associated receptor 1 (TAAR1), serotonin receptor 1b (5-HT1b), 3-T1AM, signal transduction, biased signaling, trace amines

## Abstract

Trace amine-associated receptors (TAARs) belong to the class A G-protein-coupled receptors (GPCR) and are evolutionary related to aminergic receptors. TAARs have been identified to mediate effects of trace amines. TAAR1 signaling is mainly mediated via activation of the G_s_/adenylyl cyclase pathway. In addition to classical trace amines, TAAR1 can also be activated by the thyroid hormone derivative 3-iodothyronamine (3-T1AM). Pharmacological doses of 3-T1AM induced metabolic and anapyrexic effects, which might be centrally mediated in the hypothalamus in rodents. However, the observed anapyrexic effect of 3-T1AM persists in *Taar1* knock-out mice which raises the question whether further GPCRs are potential targets for 3-T1AM and mediate the observed physiological effect. Anapyrexia has been observed to be related to action on aminergic receptors such as the serotonin receptor 1b (5-HT1b). This receptor primarily activates the G_i/o_ mediated pathway and PLC signaling through the G_β_γ of G_i/o_. Since the expression profiles of TAAR1 and 5-HT1b overlap, we questioned whether 3-T1AM may activate 5-HT1b. Finally, we also evaluated heteromerization between these two GPCRs and tested signaling under co-expressed conditions. In this study, we showed, that 3-T1AM can induce G_i/o_ signaling through 5-HT1b in a concentration of 10 μM. Strikingly, at 5-HT1b the ligand 3-T1AM only activates the G_i/o_ mediated reduction of cAMP accumulation, but not PLC activation. Co-stimulation of 5-HT1b by both ligands did not lead to additive or synergistic signaling effects. In addition, we confirmed the capacity for heteromerization between TAAR1 and 5-HT1b. Under co-expression of TAAR1 and HTR1b, 3-T1AM action is only mediated via TAAR1 and activation of 5-HT1b is abrogated. In conclusion, we found evidence for 5-HT1b as a new receptor target for 3-T1AM, albeit with a different signaling effect than the endogenous ligand. Altogether, this indicates a complex interrelation of signaling effects between the investigated GPCRs and respective ligands.

## Introduction

The trace amine associated receptor 1 (TAAR1) belongs to the class A G-protein-coupled receptors (GPCR) and is evolutionary related to the aminergic receptors (Krautwurst, 2008). TAAR1 activation leads to G_s_ signaling and is presumed to be involved in neurocognition and neurodegeneration (Zucchi et al., 2014; Pei et al., 2016). The receptor is activated by biogenic trace amines such as β-phenylethyl amine (PEA), tyramine (TYR) and octopamine (OA) (Borowsky et al., 2001; Lindemann and Hoener, 2005). TAAR1 was also found to be activated by amphetamines (Bunzow et al., 2001; Harkness et al., 2015), suggesting its involvement in methamphetamine-induced hypothermia (Miner et al., 2017). In addition to trace amines and synthetic ligands, the thyroid hormone derivative 3-iodothyronamine (3-T1AM) is another agonistic ligand for TAAR1 (Scanlan et al., 2004; Kleinau et al., 2011).

Interestingly, 3-T1AM is a promiscuous ligand as it binds to other GPCRs such as TAAR5 (Dinter et al., 2015b), the β2-adrenoreceptor (ADRB2) (Dinter et al., 2015a) and the α2A-adrenoreceptor (ADRA2A) (Dinter et al., 2015a). One of the peculiar functions of pharmacological doses, is its inducing of anapyrexia (Gachkar et al., 2017), which is very different to the classical actions of thyroid hormones (Scanlan et al., 2004; Gachkar et al., 2017). However, in mice with targeted deletion of *Taar1*, the anapyrexic 3-T1AM effect persisted (Panas et al., 2010), indicating that this effect is not mediated via TAAR1 exclusively and that other 3-T1AM targets must be proposed to mediate the anapyrexic affect. Aminergic receptors are potential candidates for 3-T1AM action. It should be of note that TAAR1 arose from gene duplication of serotonin receptors (Lindemann et al., 2005) and, as a consequence, comparing it with other aminergic receptors showed strong similarities in the ligand binding pocket (Kleinau et al., 2011).

We aimed to answer two questions: Firstly, whether serotonin receptors might be targets that could explain 3-T1AM ligand induced anapyrexic action in *Taar1* knock-out mice, given the incompletely clarified physiological role of the TAAR1 ligand 3-T1AM (Panas et al., 2010). While the full functionality of 5-HT1b is not yet understood, its involvement in depression-like behavior (Nautiyal and Hen, 2017), mediation of aggression and impulse control (Bouwknecht et al., 2001) as well as induction of anapyrexia (Bouwknecht et al., 2002) are known. Therefore, we tested the signaling properties of this receptor in interplay with the TAAR1 ligand 3-T1AM.

Secondly, we asked whether TAAR1 can interact with the serotonin receptor 1b (5-HT1b) in heteromeric complexes. TAAR1 and 5-HT1b are co-expressed in the hypothalamus (Pazos and Palacios, 1985; Borowsky et al., 2001; Lindemann et al., 2008), particularly in the preoptic area as the center of body temperature regulation. Such interaction would have an impact in the case of 3-T1AM action on both receptors and it was our aim to also decipher the signaling spectrum under receptor co-expressed conditions.

In summary, this study focused on the identification of a GPCR interaction partner for TAAR1 and at the same time provided a new aminergic human GPCR for 3-T1AM action that should be related to the anapyrexic effects induced by this ligand.

## Methods

### Cloning of plasmid constructs

**Figure 1:**
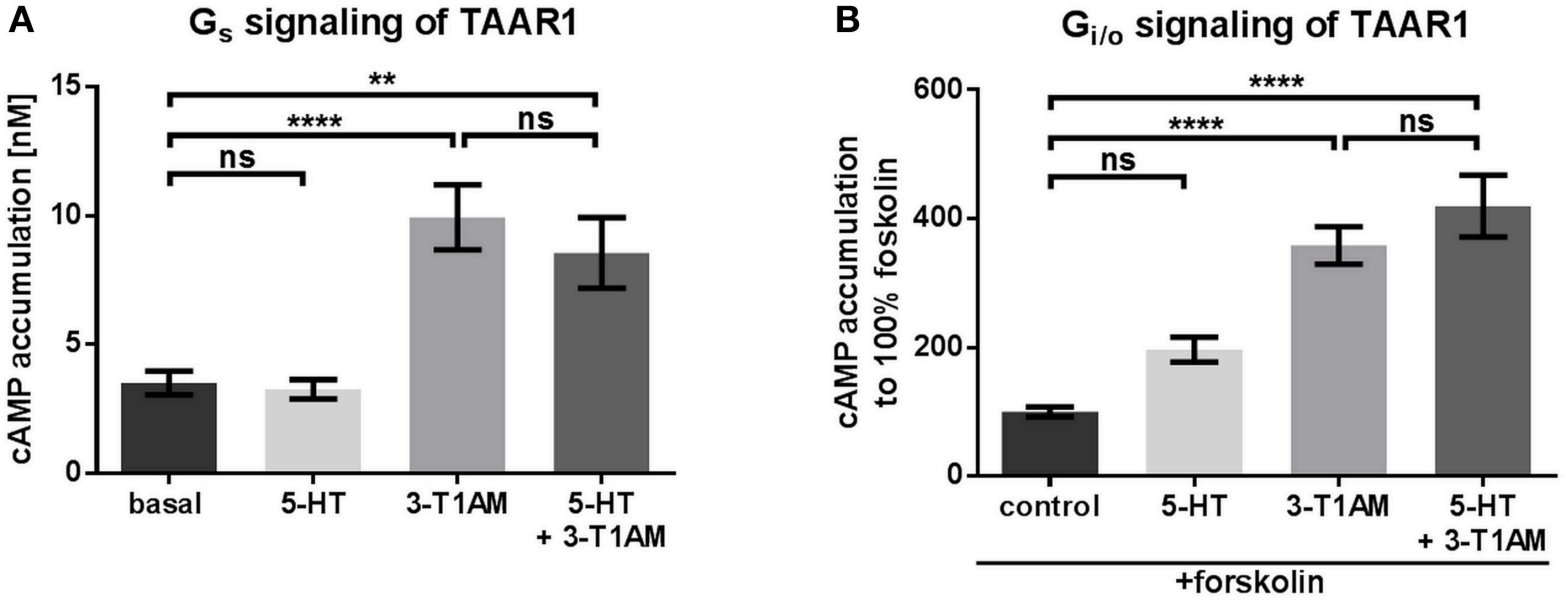
3-T1AM, but not 5-HT induces G_s_ signaling via TAAR1 in a concentration of 10 μM. To measure G_s_ and G_i/o_, the cAMP content was determined via AlphaScreen technology. HEK293 were used to overexpress TAAR1. An empty vector was used for mock transfection, which showed no endogenous effect of 5-HT or 3-T1AM (see Supplemental Figures 1A,B). **(A)** TAAR1 was stimulated with either 5-HT or 3-T1AM or both in a concentration of 10 μM. *n* = 7 measured in triplicates. **(B)** TAAR1 was stimulated with forskolin and either 5-HT or 3-T1AM or both in a concentration of 10 μM. Results of four independent experiments performed in triplicates are shown. For statistics, a one-way ANOVA was performed and the mean of each column compared with the mean of all other columns. Statistical significance was defined as ^**^p ≤ 0.01, ^****^p ≤ 0.0001.

Melanocortin-3 receptor (MC3R), 5-HT1b, ADRA2A, and TAAR1 were amplified from genomic DNA, cannabinoid 1-receptor (CB1R) was cloned from human brain cDNA. Human Ghrelin-receptor (GHSR) cDNA was purchased from UMR cDNA Resource Center, Rolla, MO, USA. For fluorescent resonance energy transfer (FRET) measurements, cAMP and luciferase assay TAAR1 was tagged at the N-terminus with the first nine amino acids of the β2-adrenoreceptor (ADRB2), resulting in βTAAR1 which according to Barak et al. (2008) enhances protein expression; subsequently, this will be referred to as TAAR1. Barak et al. showed in their studies that modification only lead to a higher membrane expression of the receptor and did not alter the signaling properties, a finding which we can confirm (Figure 1, Supplemental Figure 1). For sandwich ELISA, the genes were cloned into the eukaryotic expression vector pcDps (provided by Torsten Schöneberg, University of Leipzig, Germany). Constructs were N-terminally tagged with a hemagglutinin epitope (NHA, 50-YPYDVPDYA-30) or C-terminally tagged with a Flag epitope (C-Flag, 50-DYKDDDDK-30). The same plasmids were also used for cAMP and luciferase activity measurements. Only βTAAR1 was cloned without further tags into the expression vector pcDps for cAMP and luciferase assays. For FRET, the cDNAs were cloned into pECFP-N1 and pEYFP-N1 (Clontech Laboratories, USA). Plasmids were sequenced and verified by BigDye-terminator sequencing (PerkinElmer, Inc., Waltham, MA, USA), using an automatic sequencer (ABI 3710xl; Applied Biosystems).

### Cell culture and transfection

Cells were grown at 37°C in humidified air containing 5% CO2. For sandwich ELISA, COS7 cells were cultured in Dulbecco's modified Eagle's medium (DMEM, Biochrom GmbH, Berlin, Germany), supplemented with 10% fetal calf serum (FCS). 1 × 10^6^ cells were seeded in 6 cm dishes and transfected with Metafectene according to the manufactures protocol (Biontex, Munich, Germany).

HEK293 were cultured in MEM (Biochrom GmbH, Berlin, Germany) with 5% FCS and non-essential amino acids (Biochrom AG, Berlin, Germany). For FRET experiments HEK293, cells (1 × 10^5^ cells/ Ø 35 mm dish) were seeded in poly-L-lysine coated FluoroDishes (World Precision Instruments, Sarasota, FL, USA). Twenty-four hours later, the cells were transfected with 1 μg DNA per well and 4.5 μl GeneJuice (EMP Millipore Corp., Billerica, MA, USA). For cAMP and nuclear factor of activated T-cells luciferase (NFAT-luc) measurements, HEK293 cells were seeded in poly-L-lysine coated (Biochrom GmbH, Berlin, Germany) 96-well plates (1.5 × 10^4^ cells/well). Transient transfection of HEK293 cells was performed 24 h after seeding in supplement-free Advanced MEM (Life Technologies, Carlsbad, CA, USA), using Metafectene with 0.45 μg DNA per well and 0.45 μL Metafectene per well.

### Determination of receptor oligomerization by sandwich elisa and fluorescence resonance energy transfer (FRET)

The sandwich ELISA was performed as previously described by Piechowski et al. (2013) to investigate protein-protein interaction. COS7 cells were used since they are more robust than HEK293 and withstand the washing protocol better. An NHA tagged TAAR1 was co-transfected with a C-Flag tagged 5-HT1b or an NHA tagged 5-HT1b was co-transfected with a C-Flag tagged TAAR1, further referred to as reversely tagged. As tags had no influence on the results, data was pooled. As positive control, the growth hormone secretagogue receptor (GHSR) homodimer was used (Kern et al., 2012), while the rat muscarinic 3 receptor (rM3R) served as the non-interacting protein (NIP). Single transfection of the NHA tagged receptor was used as negative control. Cells were lysed 48 h after transfection and transferred to an anti-Flag-M2 (Sigma-Alderich, St. Louis, MO, USA) coated plate. Thereafter, a biotin-labeled anti-HA-MAB (Roche) was added for 2 h. Color reaction was carried out by using a streptavidin-labeled HRP (BioLegend, San Diego, CA, USA) to convert o-phenylendiamine. Absorption was measured at 450 nm with correction at 620 nm using an Anthos Reader 2001 (AnthosLabtech Instruments, Salzburg, Austria). Protein concentrations were analyzed with the Biuret method using a BCA Protein Assay Kit (ThermoFisher Scientific, Inc., Waltham, MA, USA) in accordance with the manufacturer's protocol.

FRET was used to confirm the dimerization result of the Sandwich ELISA. Acceptor bleaching was done as previously described (Tarnow et al., 2008). A CFP tagged receptor and an YFP tagged receptor were co-transfected including *vice versa* experiments. MC3R and GHSR were used as positive control (Rediger et al., 2011), MC3R and CB1R as negative and rM3R as NIP. Cells which expressed CFP and YFP-tagged receptors were chosen 72 h after transfection. Subsequently, YFP was photo-bleached at 512 nm 0.5 s for 10 cycles, followed by 20 cycles of bleaching and measuring CFP and YFP. The increased CFP-emission was measured at excitation at 410 nm for 0.5 s. FRET efficiency was calculated as follows: E = (F_CFPmax_ – F_CFPmin_)/F_CFPmax_. A stable protein-protein interaction is defined by a FRET efficiency between 8 and 25% (Rediger et al., 2009).

### Detection of G_s_ and G_i/o_ signaling via cAMP accumulation

G_s_ and G_i/o_ signaling were determined by measuring cAMP accumulation using the AlphaScreen technology (PerkinElmer Life Science, Boston, MA, USA) as previously described (Kleinau et al., 2011). MC3R was used as NIP. 48 h after transfection, stimulation was performed by using a stimulation buffer (138 mM NaCl, 6 mM KCl, 1 mM [MgC${\text{l}}_{2}^{*}$6H_2_O], 5.5 mM glucose, 20 mM HEPES, 1 mM [CaC${\text{l}}_{2}^{*}$2H_2_O], 1 mM IBMX). For G_s_ signaling, cells were incubated for 45 min with either 3-T1AM (Santa Cruz Biotechnology, Dallas, TX, USA), serotonin (5-HT, Sigma-Alderich, St. Louis, MO, USA) or both in a concentration of 10 μM. For G_i/o_ pathway examination, cells were co-stimulated with 50 μM forskolin (FSK, AppliChem GmbH, Darmstadt, Germany) to activate the adenylyl cyclase. Substance incubation was done at 37°C with 5% CO2 and stopped after 20 min by discarding the medium. Cells were lysed at 4°C on a shaking platform. Intracellular cAMP accumulation was determined by a competitive immunoassay based on the AlphaScreen assay kit and measured using a Berthold Microplate Reader (Berthold Technologies GmbH & Co. KG, Bad Wildbad, Germany). If cAMP values exceeded the possible measurement range, samples were diluted and the dilution factor afterwards incorporated into the cAMP concentrations.

### Determination of PLC activation by a luciferase reporter gene assay

Phospholipase C (PLC) activation was measured via the luciferase activity of a reporter gene (NFAT-luc, Promega, Madison, USA) as previously described (Cheng et al., 2010). HEK293 were co-transfected with a plasmid containing the NFAT and firefly luc reporter gene (NFAT-luc, pGL4.33), together with either receptor or empty vector plasmid DNA (mock transfection) in an equimolar concentration (0.45 μg plasmid per well). The thyrotropin (TSH) receptor served as a positive control and was stimulated with 100 mU/ml bovine TSH (Sigma-Alderich, St. Louis, MO, USA) (Winkler et al., 2010). For the PTX assay ADRA2A as Gi coupled receptor was used as positive control.

Forty-eight hours post transfection, cells were incubated for 6 h with 3-T1AM and/or 5-HT in supplement-free MEM at 37°C with 5% CO2. The media was removed and cells lysed for 15 min on a shaking platform at room temperature using 50 μl of 1x passive lysis buffer (PLB, Promega, Madison, USA). To determine luciferase activity, 10 μL sample were transferred to a black 96-well plate. Forty microliter luciferase substrate (Promega, Madison, USA) were automatically injected using the Berthold Microplate Reader and immediately measured.

### Docking of ligands into hTAAR1 and 5-HT1b structures

Our objective was to receive general and detailed insights into potential binding modes of 3-T1AM and 5-HT at the human TAAR1 and the 5-HT1b respectively. For this purpose, we used an already described TAAR1 model (Kleinau et al., 2011) that was based on the solved ADRB2 structure (Cherezov et al., 2007) and the recently solved 5-HT1b crystal structure (PDB entry 4IAQ) (Wang et al., 2013). Both inactive receptor conformations of the evolutionary related receptors were used for docking 3-T1AM and 5-HT into the conserved binding pocket of the aminergic receptors (Kleinau et al., 2011). For structure modeling, Sybyl X2.0 (Certara, NJ, US) and AMBER F99 force field for energy minimization and short molecular dynamics were used.

First, the co-crystallized ligand and the fusion protein apocytochrome BRIL (fused with the receptor ICL3) were deleted from the 5-HT1b structure. Secondly, 5-HT was placed between two known interaction points with the receptor; (i) a highly conserved aspartate (Vilar et al., 2011) in transmembrane helices (TMH) 3 (Asp129, D^3.32^ Ballesteros & Weinstein numbering Ballesteros et al., 1995), and (ii) a threonine in TMH5 (Thr213, T^5.43^) (Huang, 2003). Both putative contacts were defined as spatial constraints between ligand and receptor with a distance of 2.0 Å. This system was than energetically minimized by conjugate gradient minimization until converging at a termination gradient of 0.1 kcal/mol^*^Å. The resulting complex was refined by molecular dynamics simulations with unconstraint receptor side chains and ligand atoms (300 K, 1 ns, constrained backbone atoms of the backbone). The procedure of complex minimizations and dynamic simulations was repeated twice. Finally, the spatial distance constraints of supposed ligand receptor contacts were deleted and the complex refined by molecular dynamics (1 ns) whereby only the receptor backbone atoms were constraint, followed by energetic minimization without any constraint. The same principle protocol and initial contact points were used for docking the ligand 3-T1AM into the 5-HT1b structure.

For comparability purposes, the described general steps were also used for docking the ligands 5-HT and 3-T1AM into the hTAAR1 model, albeit with a small deviation in the initial ligand orientation. In TMH5, the putative ligand/receptor contact was initially defined to Thr194^5.42^ as the corresponding position to Thr213 of the 5-HT1b is Phe195 in TAAR1, which rules out a hydrophilic interaction of this position with the hydroxyl-group of the ligands.

### Statistical analysis

GraphPad Prism 6 (GraphPad Software Inc., La Jolla, CA, USA) was used to visualize and analyze the data. Data are indicated as mean ± standard error of the mean (SEM). Statistical analysis was performed by a one-way ANOVA, followed by Tukey's honest significant difference post hoc test. To analyze receptor co-expression, a two-way ANOVA was performed, followed by Sidak's multiple comparisons test. The statistical significance was set to ^*^p ≤ 0.05, ^**^p ≤ 0.01, ^***^p ≤ 0.001 and ^****^p ≤ 0.0001.

## Results

### TAAR1 signaling induced by different ligands

It is known that 3-T1AM stimulates G_s_/adenylyl cyclase signaling via TAAR1 (Kleinau et al., 2011). Here, we stimulated TAAR1-transfected cells with 10 μM 3-T1AM which resulted in cAMP accumulation that was significantly over basal signaling (Figure 1A, Supplemental Figure 2A). No 3-T1AM-induced signaling of cells was observed after mock transfection (Supplemental Figure 1A). In contrast to rhesus monkey TAAR1 (Xie et al., 2008), human TAAR1 can only be activated to a lesser degree in cAMP accumulation by serotonin (5-HT, Supplemental Figure 2B). It should be noted that recent reports suggest that 5-HT may activate other aminergic receptors differently to serotonin receptors, which again confirms the principle possibility of amine ligand promiscuity (Qi et al., 2017). To test whether 5-HT acts as a potential antagonist on TAAR1, co-stimulation of 3-T1AM and 5-HT in equimolar concentrations was carried out, which resulted in signaling identical to 3-T1AM stimulation alone, thus making an antagonistic activity of 5-HT at TAAR1 unlikely (Figure 1A).

TAAR1 stimulated with 10 μM 5-HT or 3-T1AM revealed no significant G_i/o_ signaling (Figure 1B), an effect that is comparable to mock transfected cells (Supplemental Figure 1B).

### 3-T1AM activates G_i/o_ signaling at the 5-HT1b

**Figure 2:**
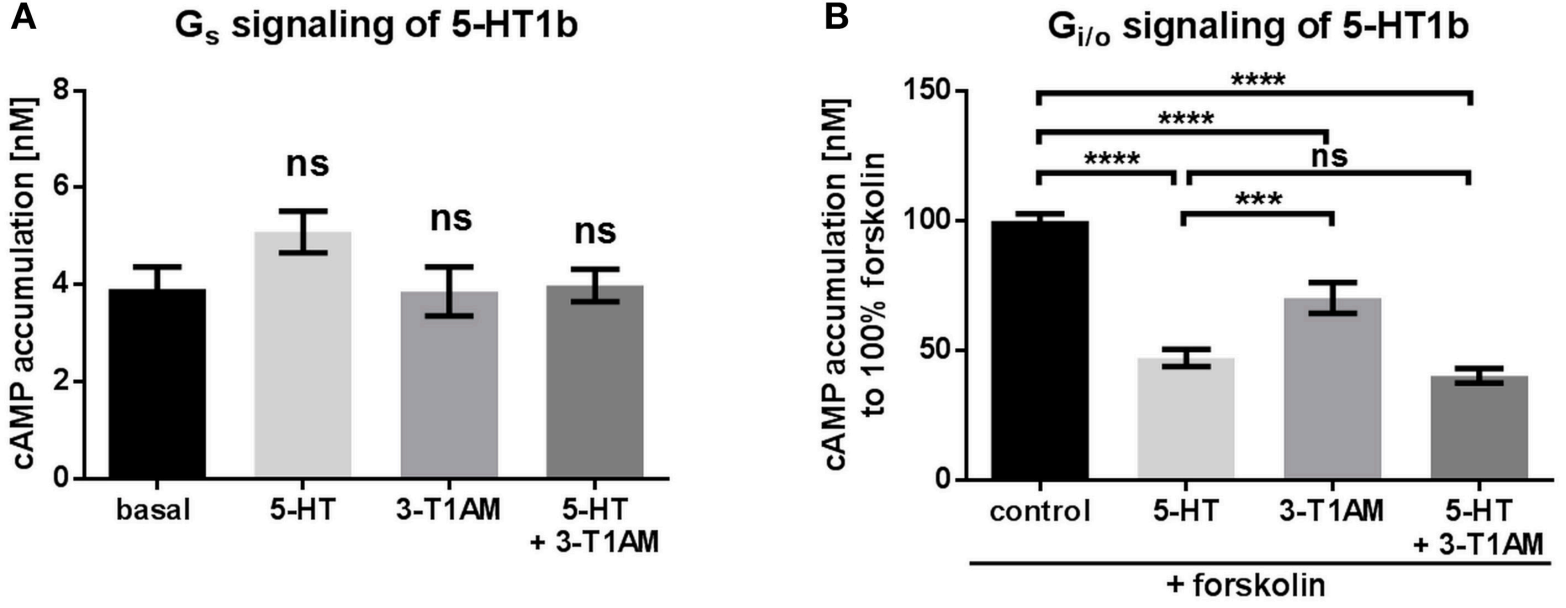
The TAAR1 agonist 3-T1AM can activated G_i/o_ signaling through 5-HT1b, but not G_s_ signaling. To measure G_s_ and G_i/o_, the cAMP content was determined via an AlphaScreen technology. HEK293 were used to overexpress 5-HT1b. An empty vector was used for mock transfection, which showed no endogenous effect of 5-HT or 3-T1AM (see Supplemental Figures 1A,B). **(A)** 5-HT1b was stimulated with either 5-HT, 3-T1AM or both in a concentration of 10 μM. *n* = 5 measured in triplicates. **(B)** 5-HT1b was stimulated with forskolin and either 5-HT, 3-T1AM or both in a concentration of 10 μM. *n* = 13 measured in triplicates. For statistics, a one-way ANOVA was performed and the mean of each column was compared with the mean of all other columns. Statistical significance was defined as ^***^p ≤ 0.001, ^****^p ≤ 0.0001.

To investigate signaling activity at 5-HT1b, the receptor-transfected cells were treated with the endogenous agonist 5-HT as well as with 3-T1AM at a concentration of 10 μM (Figure 2A). 5-HT1b is known to predominantly signal via G_i/o_ activation (Bouhelal et al., 1988). In line with this, we also did not observed any activation of G_s_ being stimulated by either 5-HT or by 3-T1AM (Figure 2A). As previously reported, 5-HT does induce a strong activation of G_i/o_ (determined as a 50% reduction of forskolin-activated adenylyl cyclase). Most remarkably, 3-T1AM also activates 5-HT1b by reducing adenylyl cyclase activity by 30% (Figure 2B). It should be noted that no synergistic effect of both ligands was observed and the reduction of cAMP accumulation is comparable to stimulation with 5-HT alone (Figure 2B).

To ensure that this effect was indeed due to G_i/o_ activation, 5-HT1b transfected cells were incubated with pertussis toxin (PTX), inhibiting G_i/o_ signaling. After pre-incubation with PTX, signaling mediated by 5-HT1b was completely blocked for both ligands (Supplemental Figure 3A).

### Different signaling properties of 5-HT and 3-T1AM at the 5-HT1b

**Figure 3:**
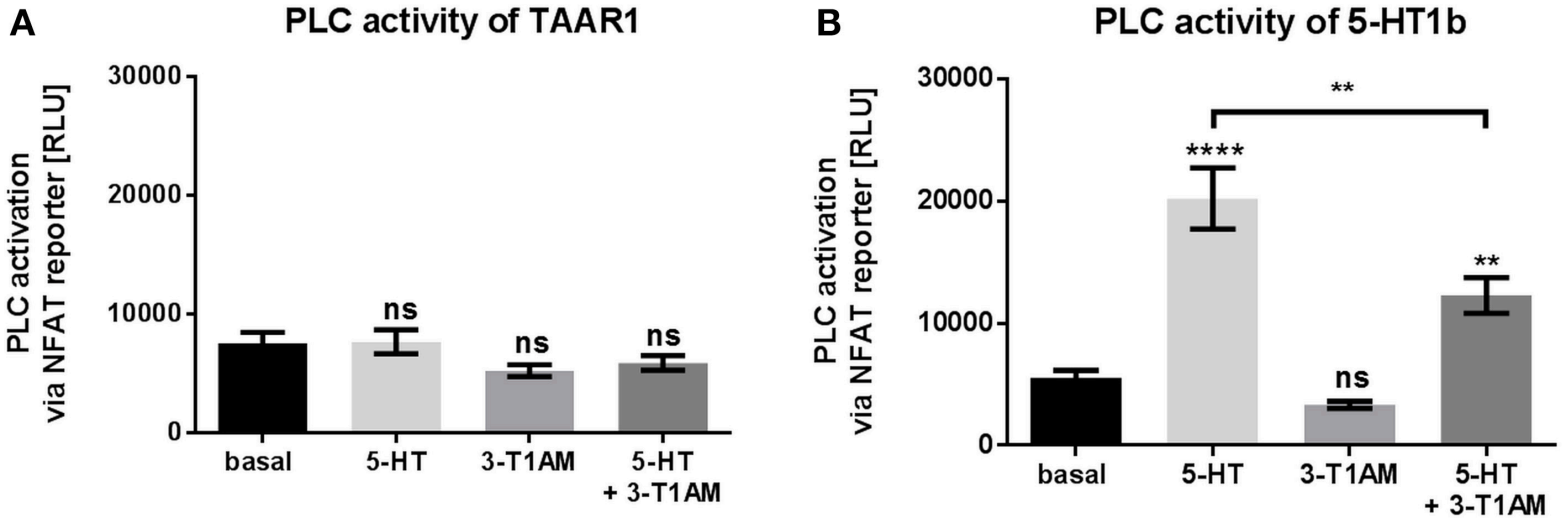
3-T1AM is not able to activate PLC via TAAR1 or 5-HT1b. Activation of PLC was measured by NFAT reporter gene assay. HEK293 were used to overexpress TAAR1 or 5-HT1b. An empty vector was used for mock transfection, which showed no endogenous effect of 5-HT or 3-T1AM (Supplemental Figures 1C). As positive control HEK293 were transfected with TSHR and stimulated with TSH (100 mU/mL) for 6 h (data ± SEM basal luciferase activity: 6931 ± 395.8 TSH stimulation: 411,660 ± 64,212). TAAR1 or 5-HT1b were stimulated with either 5-HT, 3-T1AM or both in a concentration of 10 μM for 6 h in DMEM without FCS. **(A)** TAAR1 over expression, *n* = 4 measured in triplicates. **(B)** 5-HT1b over expression, *n* = 14 measured in triplicates. For statistics, a one-way ANOVA was performed and the mean of each column was compared with the mean of all other columns. Statistical significance was defined as ^**^p ≤ 0.01, ^****^p ≤ 0.0001.

As of writing, phospholipase C (PLC) activation is not reported for TAAR1. In line with this, we also confirmed that 3-T1AM and 5-HT do not activate PLC via an NFAT reporter at TAAR1 (Figure 3A). In addition, the mock transfected cells showed no activation of PLC upon 5-HT or 3-T1AM challenge (Supplemental Figure 1C).

However, 5-HT1b can activate PLC signaling pleiotropically as a secondary pathway via G_β_γ of G_i/o_ (Dickenson and Hill, 1998; Berg and Clarke, 2001). For this reason, we tested PLC signaling of 5-HT and 3-T1AM at 5-HT1b and could confirm that 5-HT induced PTX-sensitive PLC activation 3.7-fold; however, no PCL signaling was detected for 3-T1AM (Figure 3B, Supplemental Figures 3B,C). This finding classifies 3-T1AM as a biased ligand at the 5-HT1b. This is also supported by the co-stimulation results where the binding of 3-T1AM to 5-HT1b reduces the PLC activation through 5-HT to only 2.2-fold (Figure 3B).

### TAAR1 and 5-HT1b can constitute heteromeric complexes

**Figure 4:**
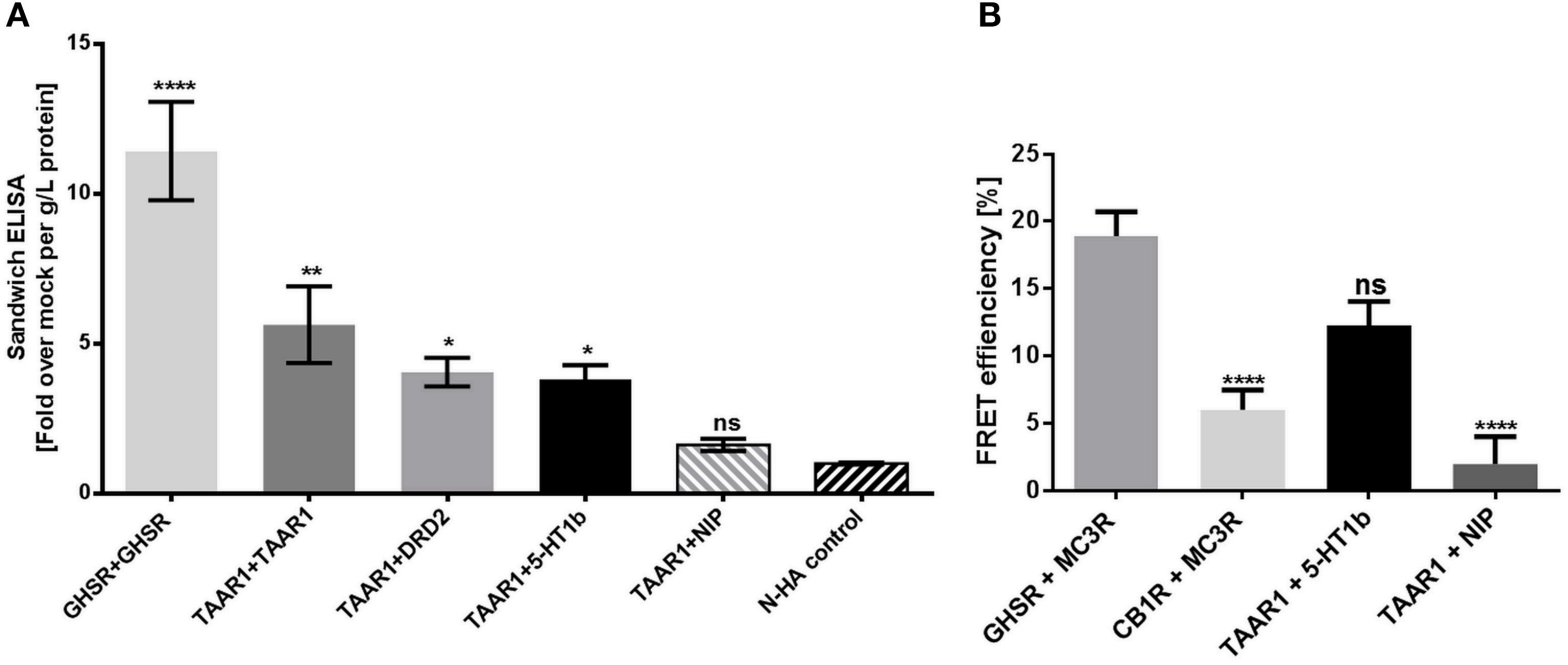
TAAR1 and 5-HT1b can interact. Sandwich ELISA and FRET were performed to investigate dimerization. **(A)** For sandwich ELISA, receptor pairs were overexpressed in COS-7 cells, receptors were NHA- and FLAG-tagged and *vice versa*. The homodimers GHSR and TAAR1, as the heterodimer TAAR1 and DRD2 were used as positive control; TAAR1 and rM3R were used as negative control. NHA control are cells over expressing NHA-tagged TAAR1, 5-HT1b, DRD2, and GHSR. Data are shown as mean ± SEM of fold over mock transfection. Data were pooled from four to eight independent assays measured in three replicates. For statistical analysis, a one-way ANOVA was performed in relation to mock transfection. **(B)** To determine FRET efficiency, HEK293 overexpressed receptor pairs, one being CFP and one the YFP tagged and *vice versa*. The heterodimer GHSR and MC3R was used as positive control, CB1R and MC3R as negative control. rM3R was used as NIP for TAAR1. Compared to GHSR and MC3R dimers, the negative control and TAAR1 with the NIP showed no protein-protein-interactions, whereas TAAR1 and 5-HT1b co-expression did. Data were pooled from 11 to 19 cells and a one-way ANOVA was performed and the mean of each column was compared with the mean of all other columns. Statistical significance was defined as ^*^p ≤ 0.05, ^**^p ≤ 0.01, ^****^p ≤ 0.0001.

We tested for a potential TAAR1/5-HT1b interaction or close spatial distance between the receptors with two independent methods. Determination of receptor heteromers with differed N- and C-terminally tagged receptors was performed for TAAR1 and 5-HT1b. In comparison to the TAAR1/D2R heteromer, TAAR1 interaction with 5-HT1b is at the same level and stronger in relation to the negative control (Figure 4A). To verify obtained data by means of a second method, we applied FRET measurements. We demonstrated an interaction of TAAR1 and 5-HT1b as the FRET efficiency equals that of the already described heteromer GHSR/MC3R (Figure 4B; Rediger et al., 2009), while the negative control and TAAR1 combined with an non interacting receptor have significantly lower FRET efficiencies.

To obtain deeper insights into the functional properties of this indicated putative interaction, we tested signaling properties of each receptor as individually transfected into cells, co-expressed and by stimulation with their respective ligands.

### Co-expression and Co-stimulation of TAAR1 and 5-HT1b results in a modified signaling outcome

**Figure 5:**
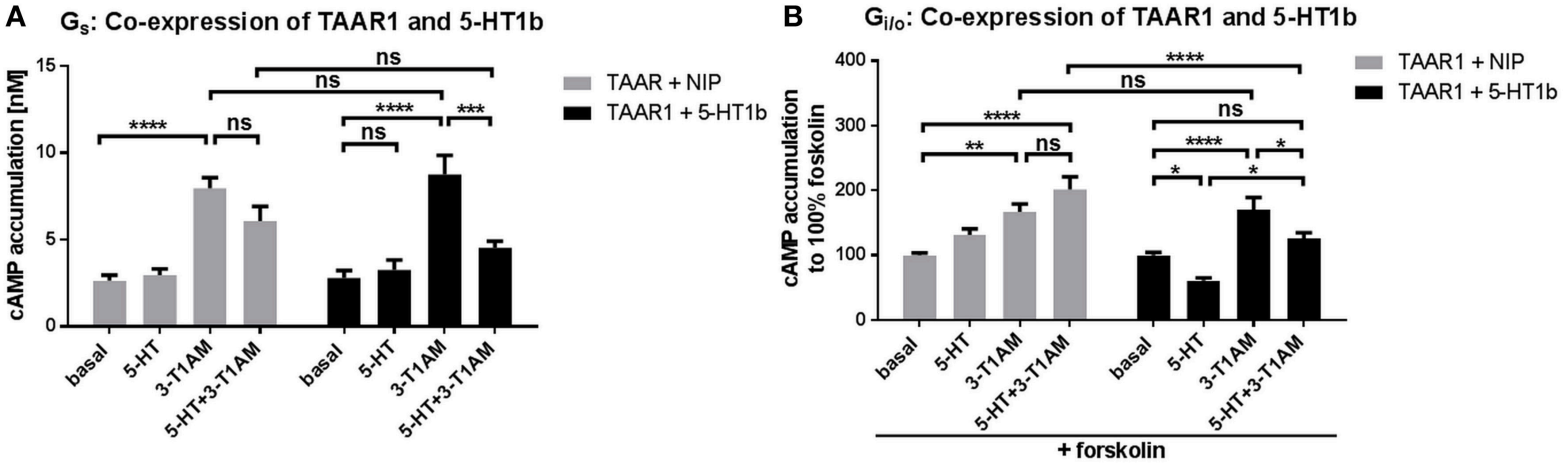
Modification of signaling properties of 3-T1AM at the TAAR1/5-HT1b heteromer. To measure G_s_ and G_i/o_ activation, cAMP accumulation was measured via an AlphaScreen technology. For control, HEK293 expressing TAAR1 and a non-interacting protein (MC3R) were used (gray bars) and compared with TAAR1 and 5-HT1b co-expression (black bars). 3-T1AM does not induce G_i/o_ activation in the heteromeric constellation and the effect of 5-HT is lessened. Data were pooled from 6 to 10 independent assays and measured in triplicates. For statistics, a one-way ANOVA was performed to compare the ligands, a two-way ANOVA was used to compare TAAR+NIP and TAAR+5-HT1b and considered significant with ^*^p ≤ 0.05, ^**^p ≤ 0.01, ^***^p ≤ 0.001, ^****^p ≤ 0.0001. **(A)** Cells were stimulated with either 5-HT, 3-T1AM or both in a concentration of 10 μM. **(B)** For G_i/o_, cells were additionally co-stimulated with forskolin and the ligands.

3-T1AM application at cells with co-expressed TAAR1 and 5-HT1b resulted in G_s_ signaling levels comparable to TAAR1 expression alone (Figure 5A). Additionally, co-stimulation by 3-T1AM and 5-HT in cells with co-expressed receptors, led to reduced levels of G_s_ mediated signaling which is caused by G_i/o_ activation through 5-HT and 3-T1AM at the 5-HT1b.

Interestingly, when TAAR1 and 5-HT1b are co-expressed, 3-T1AM only exerts G_s_ activation, as is indicated by comparable signaling activity of 3-T1AM for TAAR1 + NIP and TAAR1 + 5-HT1b (Figure 5B). This finding indicates that the G_i/o_ signaling effect of 3-T1AM at the 5-HT1b is lost in the heteromeric constellation. Furthermore, the effect of 5-HT at the heteromer is not as strong as when 5-HT1b is expressed alone (FSK-induced cAMP amount reduced by 40% for TAAR1/5-HT1b and by 50% for 5-HT1b). PLC activation was not altered through co-expression of TAAR1 and 5-HT1b (Supplemental Figure 3D).

### Putative interactions and binding modes of 3-T1AM and 5-HT with TAAR1 and 5-HT1b

**Figure 6:**
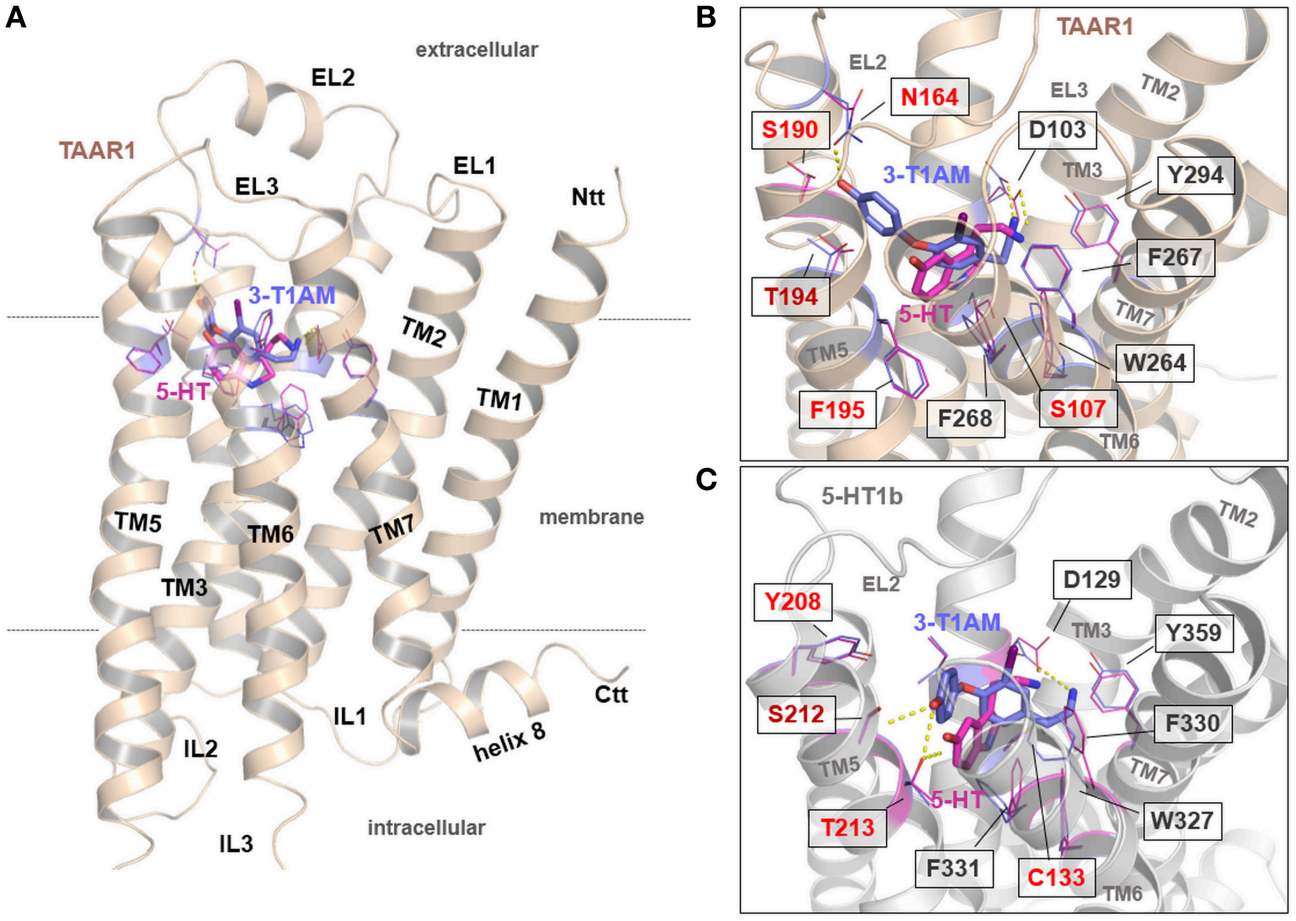
General and detailed insights into potential binding modes of 3-T1AM and 5-HT at the hTAAR1 and the 5-HT1b. **(A)** This backbone illustration visualizes the general architecture of TAAR1. The principle ligand binding site and preferred ligand/receptor contact points are known (e.g., Cherezov et al., 2007; Tan et al., 2008; Vilar et al., 2011; Warne et al., 2011; Wichard et al., 2011). **(B,C)** Comparison of receptor side chains in both receptors covering the ligand binding-pockets revealed that essential residues are conserved, however, small deviations in the amino acid constitution of the binding pockets might be of significance (indicated by different coloring). 5-HT binds into TAAR1 in a comparable mode as supposed for 5-HT in the 5-HT1b. **(B,C)**. In the 5-HT1b/5-HT complex the ligand interacts with Thr213 in TMH5 via hydrogen bonding, which cannot be observed for 5-HT in TAAR1. It should be noted that 3-T1AM binds to the 5-HT1b in a nearly comparable way as was found for 5-HT **(C)** In TAAR1, the ligand 3-T1AM is located between the Asp103 in TMH3 and the aromatic moiety is located between TMH3-4-5, whereby according to these supposed complexes the hydroxyl-group interacts with Asn164 at the transition between the EC2 and TMH4 **(B)**. This is vastly different to 3-T1AM binding in the 5-HT1b **(C)**, which is reasoned by amino acids Ser190 (TAAR1) and Tyr208 (5-HT1b) at corresponding positions in TMH5. While the bulky aromatic ring of Tyr208 in 5-HT1b blockades the orientation of ligands between TMH3-4-5, in TAAR1 this region is accessible for the ligand because of the smaller serine side chain.

The principal ligand binding site and ligand/receptor contact point for aminergic receptors are well known from determined crystal structures, bioinformatic approaches and mutagenesis studies (e.g., Cherezov et al., 2007; Tan et al., 2008; Vilar et al., 2011; Warne et al., 2011; Wichard et al., 2011). Therefore, we initially placed 5-HT and 3-T1AM into the empty putative binding side, located between the extracellular loops (EL) EC2 and transmembrane helices (TMH) 3, 5, 6, and 7 (Figure 6A) of the TAAR1 model and the 5-HT1b crystal structure, while taking known ligand/receptor contact points as constraints into consideration (see section Materials and Methods). These structural complexes where refined by short-time molecular dynamics and energetically optimized by energy minimization. The resulting models provided information on the putative binding modes of both ligands into these receptors. Comparison of the receptor side chains covering the ligand binding-pockets revealed that essential residues are conserved between these aminergic receptors. However, small deviations can also be observed and are relevant for the differences observed for ligand binding contacts (Supplemental Figure 4).

5-HT binds into TAAR1 in a comparable mode as is supposed for 5-HT in 5-HT1b (Figures 6B,C). However, in the 5-HT1b/5-HT complex, the ligand interacts with Thr213 in TMH5 via a hydrogen bond, which cannot be observed for 5-HT in TAAR1. This can be explained by a deviation in the amino sequence at this position. In TAAR1, a phenylalanine is located at the corresponding position (Phe195) and thus a hydrogen bonding with the ligand hydroxyl-group cannot be established. It could be that this interaction between TAAR1 and 5-HT alternatively occurs with T194 (adjacent amino acid) which is consecutive to Phe195. In conclusion, besides a conserved ligand orientation of 5-HT in TAAR1 and 5-HT1b, the essential interaction is different between both binding poses.

3-T1AM binds to the 5-HT1b in an almost comparable way to 5-HT (Figure 6C), albeit with a small structural shift that is likely due to the larger dimension of 3-T1AM.

The most significant difference in ligand binding can be observed between the complexes 3-T1AM/TAAR1 and 3-T1AM/5-HT1b. In TAAR1, the ligand 3-T1AM is located between the TMH3 (anchored at Asp103) and the aromatic moiety is oriented between TMH3, TMH4 and TMH5, whereby the hydroxyl-group potentially interacts with asparagine 164 at the transition between the EC2 and TMH4 (Figure 6B and Supplemental Figure 4). This stands in strong contrast to the 3-T1AM binding in 5-HT1b (Figure 6C). The explanation for this can be found in the two different amino acids Ser190 (TAAR1) and Tyr208 (5-HT1b) at the corresponding positions in TMH5. While the aromatic hydroxyphenyl ring of Tyr208 in 5-HT1b spatially blockades the orientation of the aromatic phenyl ring of the ligand, this region is accessible for the ligand in TAAR1 as the serine side chain is short and not bulky like the corresponding tyrosine. Therefore, these studies suggest an additional or newly formed hydrogen bond between 3-T1AM and TAAR1 (with Asn164 in the EC2) as opposed to the putative binding mode with 5-HT1b.

## Discussion

Our aim in this study was to elucidate whether 5-HT1b is a potential 3-T1AM target. We wanted to verify the hypothesis that an interplay between TAAR1 and other aminergic receptors like the 5-HT1b should modify signaling properties as is already known from other heteromeric GPCRs (Rozenfeld and Devi, 2011). For several assays, we used an N-terminally tagged TAAR1. Besides GPCR trafficking, the N-terminus is relevant for ligand binding, signal transduction and even receptor interaction, with point mutations and deletions predominantly leading to reduced receptor functionality (Uddin et al., 2012; Belmer et al., 2014; Coleman et al., 2017). However, nearly all class A GPCRs possess a short N-terminus and we did not observe that an extension of the ADRB2 with nine amino acids lead to a different signaling of TAAR1 (Figure 1A, Supplemental Figure 2A). Additionally, both interaction assays purpose a protein-protein-interaction between TAAR1 and HTR1b, even though an unmodified TAAR1 was used for the sandwich ELISA and a tagged one for FRET.

### Biased signaling of 3-T1AM at the 5-HT1b

It is significant that 3-T1AM can modulate 5-HT1b signaling as reported here (Figure 2), since this induced signal is biased when compared to 5-HT action on this receptor in terms of PLC activation by G_β_γ. It is known that the heterotrimeric G_i/o_ protein can remain as a complex of G_α_ and G_β_γ after activation (Bunemann et al., 2003; Frank et al., 2005). This finding leads to the question what physiological effects might be influenced by 3-T1AM action on this aminergic receptor, particularly in terms of a particular signaling pathway. The 5-HT related hormone system is evolutionary ancient and has important functions in lower animals, even as it is involved in behavioral tasks such as egg laying (Nichols and Sanders-Bush, 2001). In higher animals, 5-HT is involved in a variety of complex behaviors and processes such as aggression, sleep, appetite and mood (Nichols and Sanders-Bush, 2001). 5-HT-induced effects are usually mediated by a group of receptors with 14 members (Hoyer et al., 1994). Specific targeting of 5-HT-receptors for treatment of a diverse set of pathophysiological conditions such as depression, schizophrenia, migraine among others is an important issue for which the functional selectivity at these receptors comes into play (Bohn and Schmid, 2010).

For the first time, our study showed that 3-T1AM can activate 5-HT1b in terms of G_i/o_ coupling, but also that the induced signaling profile of 3-T1AM is different for 5-HT as it lacks induction of PLC signaling via G_β_γ of G_i/o_. As of writing, 3-T1AM is known to be involved in various physiological functions (Zucchi et al., 2014) such as thermoregulation, regulation of glucose homeostasis (Manni et al., 2012), modulation of pain and memory (Manni et al., 2013) as well as effects on sleep and motor activity (James et al., 2013). After conversion of 3-T1AM to 3-iodothyroacetic acid, modulation of the histaminergic system (Laurino et al., 2015) and vasodilatation (Gachkar et al., 2017) was observed. It should be hypothesized that some of these effects could potentially be due to the activation of 5-HT1b rather than being related to the 3-T1AM target TAAR1. However, differential signaling of 3-T1AM at 5-HT1b provides an opportunity to selectively activate the 5-HT1b in terms of a specific pathway. Further studies should reveal whether 3-T1AM is also capable of modifying the function of other serotonin receptors and if the action of 3-T1AM at 5-HT-receptors is involved in thermoregulation.

We further asked how these two ligands interact with their respective receptors and how far their binding modes differ or overlap. For this purpose, we used available structural information and performed molecular dynamic simulations (Figure 6 and Supplemental Figure 4). The supposed ligand bound receptor complexes suggest three main results: (1) 5-HT can bind with a near identical orientation into both receptors, whereby small differences between the receptors in the amino acids of TMH5 are likely associated with slight deviations in the putative intermolecular hydrogen bonds. (2) 5-HT is located partially different in TAAR1 compared to 3-T1AM, which can be explained by differences in the ligand dimensions and intermolecular contacts to TMH5 which could also explain why this ligand induces lower G_s_ mediated signaling at this receptor. (3) Moreover, while 3-T1AM can be characterized by a comparable binding mode in the 5-HT1b as supposed for 5-HT, which is in line with the G_i/o_ activation by 3-T1AM at this receptor identified in our study, binding of this ligand into the TAAR1 is significantly different. This is likely due to the specific amino acid composition surrounding the ligand binding site (Figures 6B,C). In contrast to the orientation of 3-T1AM in 5-HT1b, 3-T1AM in TAAR1 is orientated more toward TMH4 and the EC2, which can be explained by different amino acids in TMH5 covering the ligand binding pockets (Figure 6B). In conclusion, these molecular insights on one hand support similarities in the investigated receptor/ligand complexes, but on the other hand also highlight potential differences between agonistic ligands in aminergic receptors and their associated diverse effects on signaling.

### Co-expression of TAAR1 and 5-HT1b in interplay with 3-T1AM and 5-HT modulates the resulting signaling profile

By using two independent methods, sandwich ELISA and FRET, we confirmed that TAAR1 has the potential to interact with the 5-HT1b (Figure 1). This finding extends the physiologically possible interactome of TAAR1 and the 5-HT1b as they are both expressed in in the hypothalamus (Pazos and Palacios, 1985; Borowsky et al., 2001; Lindemann et al., 2008), in the striatum (Espinoza et al., 2011; Navailles and De Deurwaerdère, 2011) and in the dorsal raphe nuclei (Lindemann et al., 2008; Beliveau et al., 2017). As a result, it is of further interest how such potential interaction or interplay may impact the functionality of these receptors. Two general possibilities must be considered, either heteromeric interaction itself has an impact on signaling properties or the ligand mediated action is influenced by such heteromerization (George et al., 2002). Moreover, the scenario becomes even more complex if both receptors respond to the same ligands, but with diverse signaling pathways.

Therefore, we studied the potential heteromeric complex by experiments under co-expressed conditions and simultaneously tested the signaling effects by individual or by co-stimulated ligand application. Our findings on the TAAR1/5-HT1b heteromer constitution are in accordance with several other reports, pointing to the capacity of TAAR1 to interplay with diverse proteins such as the D2R (Espinoza et al., 2011; Navailles and De Deurwaerdère, 2011) or the ADRA2A (Dinter et al., 2015b).

In our study, we observed that the 5-HT and 3-T1AM mediated activation of G_i/o_ via the 5-HT1b is modified under co-expressed receptor conditions (Figure 5B). Notably, the action of 3-T1AM at the heteromer did not show any G_i/o_ activation at 5-HT1b and the effect of 5-HT is reduced. In principle, uncoupling of a TAAR1 interaction partner from its signaling pathway was observed for norepinephrine-induced ADRA2A signaling in a TAAR1/ADRA2A heteromer (Dinter et al., 2015b). The effect we observed here is different and we speculate that under the above described conditions, the G_s_ activation induced by 3-T1AM binding at the TAAR1 is oppositional to the G_i/o_ mediated effect of 5-HT1b/5-HT and 5-HT1b/3-T1AM complexes (Figure 3). This would also suggest that under co-expressed conditions (like for TAAR1 and the 5-HT1b in several tissues), the availability and number of ligands (concentrations) are regulating elements for the resulting signaling output. It was reported that for serotonin receptor ligands, the thermic effect is concentration dependent, e.g., high concentrations of 5-methoxy-*N,N*-dimethyltryptamine (5-MeODMT) induce hyperthermia, while lower doses result in anapyrexia, mediated by 5HT1b and HTR2 (Gudelsky et al., 1986).

In summary, we found in this study that 3-T1AM is a biased ligand of 5-HT1b. Further studies will have to show if this receptor is responsible for the 3-T1AM induced anapyrexic effect seen in rodents or if other thermoregulatory GPCRs are involved. Our work also underlines the complex role of TAAR1 concerning modulating signal transduction of various aminergic GPCRs and different promiscuous ligands. Targeting of receptors in this signaling network for therapeutically purposes might result in a plethora of effects depending on ligand concentration and expression profile of interacting partners in different tissues.

## Author contribution

JB: Experimental design, performed the experiments, data analysis, evaluation and discussion, wrote the manuscript, figure preparations and final approval. JD: Performed sandwich ELISA experiments, approved the manuscript. CH and MR: Data evaluation and discussion, approved the manuscript. SP: Data evaluation and discussion, wrote manuscript, the approved the manuscript. PS: Modeling and docking studies, data analysis and discussion, wrote the manuscript and final approval. MS: Modeling and docking studies, data analysis and discussion, wrote the manuscript and final approval. JM: Data evaluation and discussion, wrote manuscript and approved the manuscript. GK: Experimental design, modeling and docking studies, data analysis, evaluation and discussion, wrote the manuscript and final approval. HB: Study design, performed the experiments, data analysis, evaluation and discussion, wrote the manuscript and final approval.

### Conflict of interest statement

The authors declare that the research was conducted in the absence of any commercial or financial relationships that could be construed as a potential conflict of interest.
