# Supplementary material for: The Trace Amine-Associated Receptor 1 Agonist 3-Iodothyronamine Induces Biased Signaling at the Serotonin 1b Receptor
Source: Front Pharmacol. 2018 Mar 12;9:222. doi: 10.3389/fphar.2018.00222 (PMC5857711; doi:10.3389/fphar.2018.00222)
Supplement: Supplementary file 1 [file DataSheet1.docx]

***Supplemental material***

**THE TRACE AMINE RECEPTOR 1 AGONIST 3-IODOTHYRONAMINE INDUCES BIASED SIGNALING AT THE SEROTONIN 1B RECEPTOR**

Julia Bräunig^1^, Juliane Dinter^1^, Carolin Höfig^2^, Sarah Paisdzior^1^, Michal Szczepek^5^, Patrick Scheerer^5^, Mark Rosowski^3^, Jens Mittag^4^, Gunnar Kleinau^1,5,*^, Heike Biebermann^1,*^

**^1^** Institute of Experimental Pediatric Endocrinology, Charité – Universitätsmedizin Berlin, corporate member of Freie Universität Berlin, Humboldt-Universität zu Berlin, and Berlin Institute of Health; Berlin, Germany;

^2^ Institute of Experimental Endocrinology, Charité – Universitätsmedizin Berlin, corporate member of Freie Universität Berlin, Humboldt-Universität zu Berlin, and Berlin Institute of Health; Berlin, Germany;

^3^ Technical University of Berlin - Institute of Biotechnology, Department Medical Biotechnology;

^4^ University of Lübeck - Center of Brain Behavior and Metabolism;

^5^ Group Protein X-ray Crystallography and Signal Transduction, Institute of Medical Physics and Biophysics, Charité – Universitätsmedizin Berlin, corporate member of Freie Universität Berlin, Humboldt-Universität zu Berlin, and Berlin Institute of Health; Berlin, Germany;

* contributed equally


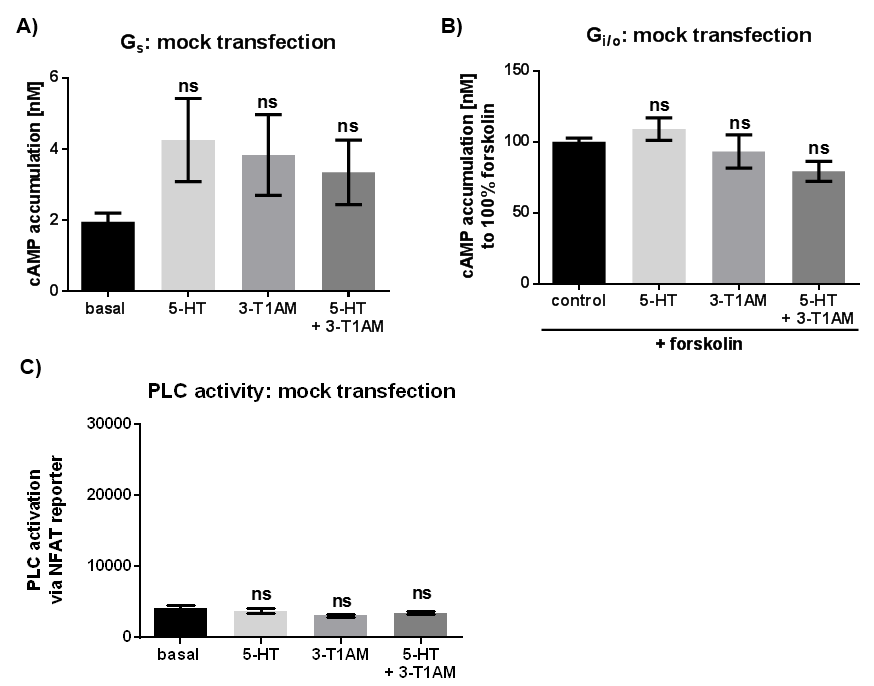
**Supplemental figure 1**

***Supplemental figure 1: 5HT and 3-T1AM have no influence on the endogenous signaling of HEK293.*** 3-T1AM influence of HTR1b is a PTX-sensitive G_i/o_ effect. To measure G_s_ and G_i/o,_ the cAMP content was measured via AlphaScreen technology **(A+B)** and for PLC activity a NFAT luciferase assay was performed **(C)**. HEK293 were transfected with an empty vector as mock control **(A, B, C)**. In all assays, cells were stimulated with either 5HT, 3-T1AM or both in a concentration of 10 µM. As statistical test, a one-way ANOVA was performed. **(A)** Data are pooled from n = 3 measured in triplicates. **(B)** Data are pooled n = 13 measured in triplicates. **(C)** Data are assessed from n = 8 measured in triplicates.

**Supplemental figure 2**


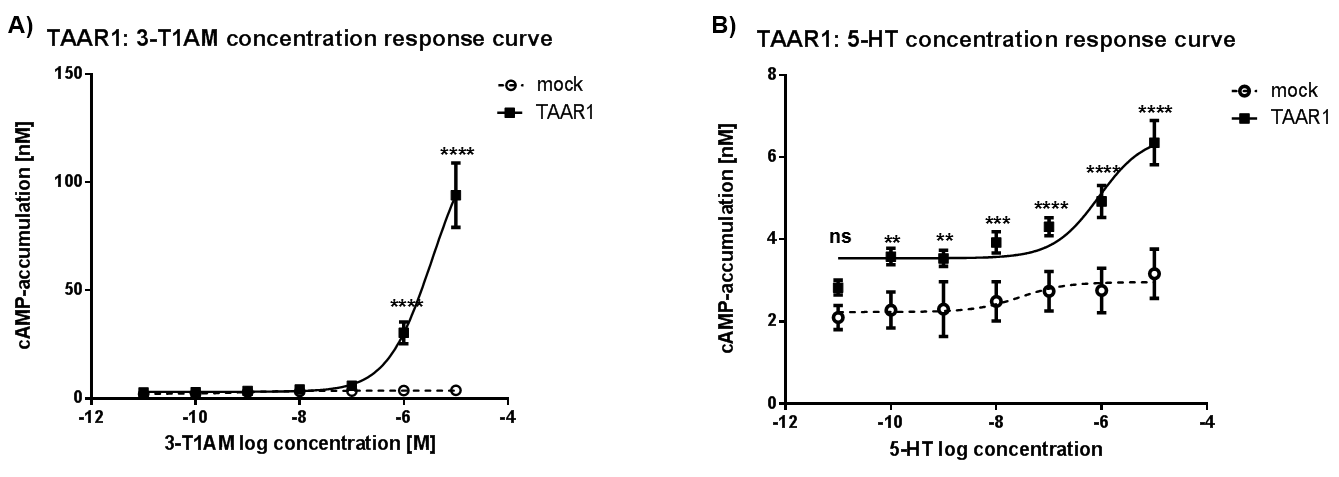


***Supplemental figure 2: 3-T1AM and 5-HT dose response curves at TAAR1.*** 5-HT only leads to a minor cAMP increase through TAAR1. To measure G_s_, the cAMP content was measured via an AlphaScreen Kit. HEK293 were transfected with an empty vector as mock control or TAAR1 **(A+B)**. In all assays, cells were stimulated with either 3-T1AM or 5HT in different concentrations between 10^-5^ and 10^-11^ M. As statistical test, a one-way ANOVA was performed. **(A)** Data are pooled from n = 5 measured in triplicates. **(B)** Data are pooled form n = 4 measured in triplicates.

**Supplemental figure 3**


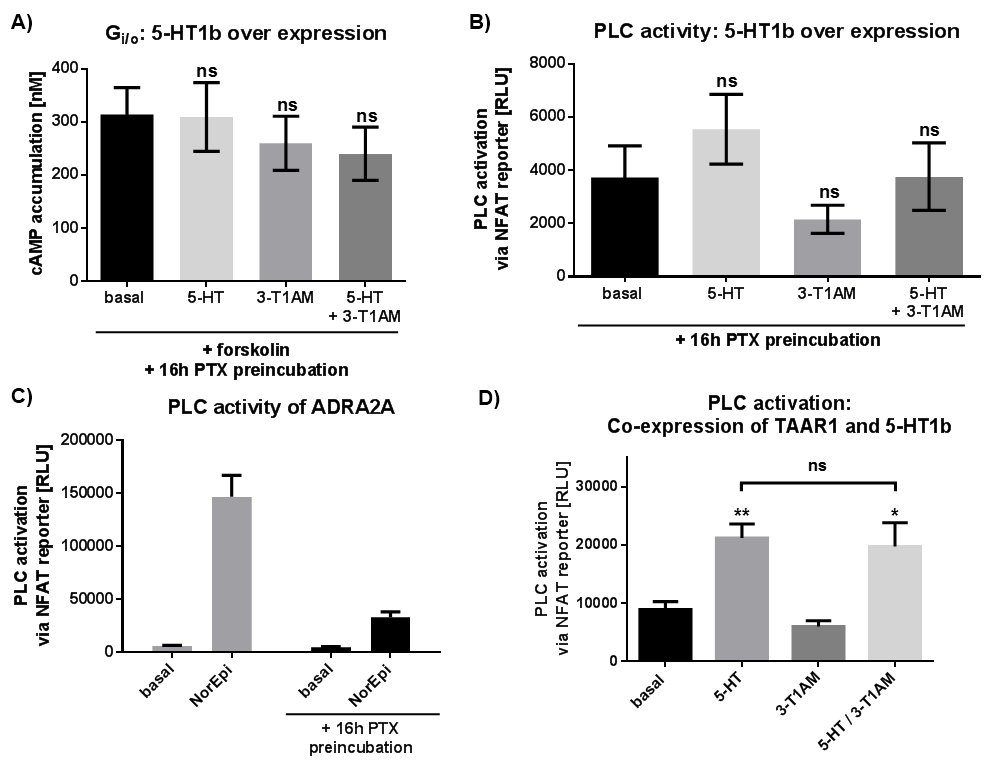


***Supplemental figure 3:* *Adenylyl cyclase and PLC activity of 5-HT1b are pertussis toxin (PTX) sensitive.*** For G_i/o_, the cAMP content was measured via AlphaScreen technology **(A)** .For PLC activity, a NFAT luciferase assay was performed **(B+C+D)**. 5-HT1b and ADRA2A was pre-incubated with PTX for 16 hours. **(A)** Samples were stimulated with forskolin and either 5-HT, 3-T1AM or both in a concentration of 10 µM. Data are indicated as mean ± SEM and are pooled from n=4 measured in triplicates. As statistical test, a one-way ANOVA was performed. **(B)** Samples were stimulated with either 5-HT, 3-T1AM or both in a concentration of 10 µM for six hours. Data are indicated as mean ± SEM and are pooled from n=3 measured in triplicates. As statistical test, a one-way ANOVA was performed. **(C)** ADRA2A was used as PTX sensitive positive control for PLC activity. Samples were stimulated with either 5-HT, 3-T1AM or both in a concentration of 10 µM for six hours. Data are indicated as mean ± SEM and are pooled from n=4 measured in triplicates. **(D)** HEK293 were transfected with 5-HT1b and TAAR1. Co-expression had no effect on PLC activity. Data are indicated as mean ± SEM and are pooled from n=4 measured in triplicates.

**Supplemental figure 4**


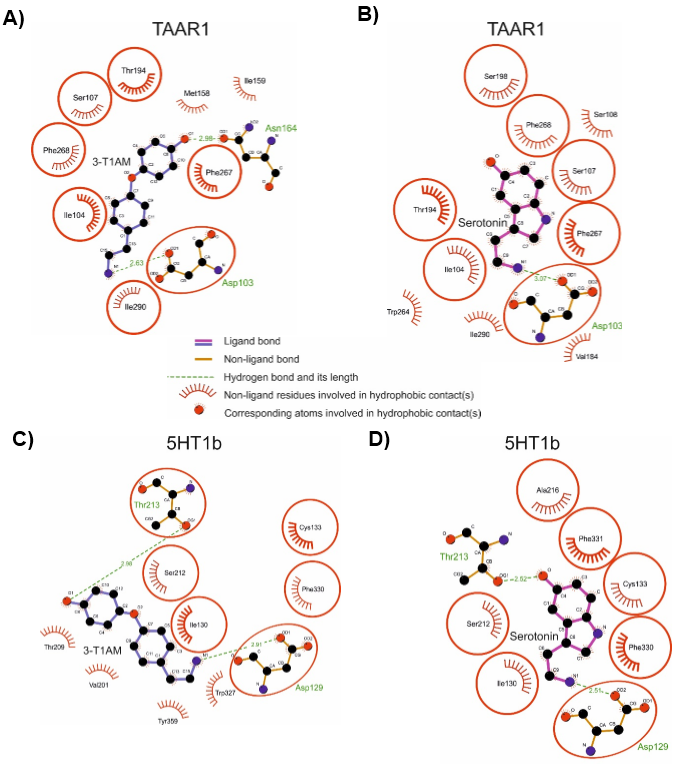


***Supplemental figure 4:* *Putative ligand-receptor interactions.*** Schematic interaction plots for the four putative receptor/ligand complexes suggested by our modeling studies (Figure 6) show a schematic overview of potential hydrogen bonding and van der Waals interactions. Potential hydrogen bonds were analyzed using *HBPLUS* as implemented in the program *LIGPLOT+ 1.45,* which was used to draw the final schematic views. Residues with distances less than 3.9 Å were considered to be in van der Waals contact.
